# Incidence, Mortality and Survival Trends in Breast Cancers Coincident with Introduction of Mammography in the Nordic Countries

**DOI:** 10.3390/cancers14235907

**Published:** 2022-11-29

**Authors:** Kari Hemminki, Asta Försti

**Affiliations:** 1 Biomedical Center and Faculty of Medicine in Pilsen, Charles University in Prague, 30605 Pilsen, Czech Republic; 2 Division of Cancer Epidemiology, German Cancer Research Center (DKFZ), Im Neuenheimer Feld 580, 69120 Heidelberg, Germany; 3 Hopp Children’s Cancer Center (KiTZ), 69120 Heidelberg, Germany; 4 Division of Pediatric Neurooncology, German Cancer Research Center (DKFZ), German Cancer Consortium (DKTK), 69120 Heidelberg, Germany

**Keywords:** mammography, periodic survival, treatment, cancer control

## Abstract

**Simple Summary:**

Mammography has been introduced in many countries for the early detection of breast tumors and thus prevention of deaths in breast cancer. Survival in breast cancer has universally improved but it is not known to what extent mammography may have contributed to this positive development. National mammographic screening was offered to women in Finland and Sweden since the 1980s and in Denmark and Norway since the 1990s. We assessed, at the national level, if relative survival had improved after the introduction of the screening activities in age groups that were screened compared to those not screened. We observed an improvement in five-year and 10-year survival after the period of national mammography screening was in place. Timing and age-specific targeting of the improvements suggest that mammography may have contributed these. However, as we had no individual data on women who used the service, more detailed studies are needed to confirm the suggested survival advantage.

**Abstract:**

Survival in female breast cancers has generally improved but the relative contribution of early detection or treatment in this positive development is not known. Our aim was to assess the possible role of national mammography screening programs in survival improvement. Such screening has been offered to women, usually at 50–69 years of age, in Finland and Sweden since the 1980s and in Denmark and Norway since the 1990s. Participation rates have been high, ranging from 60% to 90%. We analyzed incidence and mortality changes and relative 5- and 10-year survival trends in breast cancer as novel measures in these countries using the NORDCAN database. Survival trends were compared in age groups of women who were screened to those who were not screened. We observed a relative survival advantage in 5-year and 10-year survival in the screened age groups after the period of national mammography screening was in place and this was consistent in each country. Timing and age-specific targeting of the improvements suggest that mammography may have contributed to the survival benefits. However, as we had no individual data on women who used the service, more detailed studies are needed to confirm the suggested survival advantage, particularly concerning mortality in stage-specific breast cancer.

## 1. Introduction

Mammography has been the main population screening method for breast cancer in developed countries [1,2,3]. The rationale for mammography is to detect early tractable tumors and thus reduce mortality and improve survival. However, once introduced into a population, it will cause an initial increase in the observed incidence in breast cancer [1,4,5]. While the success of mammography has not been universally accepted, some but not all population-based studies from the Nordic countries have reported a mortality reduction in breast cancer by some 25–35% [4,5,6,7,8,9]. Female breast cancer is one of many cancers for which survival has improved over the past years, the reasons for which have many interpretations [10,11,12,13]. Breast cancer 5-year relative survival has increased from 60% to 90% in the Nordic countries during the past 50 years [14]. Survival in a particular cancer is influenced by numerous factors, ranging from demographics (age, sex, social background) and cancer related (stage, grade) factors to treatment and overall care. Obviously, participation in effective screening methods could contribute to a survival advantage.

We assessed here relative survival in female breast cancer from Denmark (DK), Finland (FI), Norway (NO), and Sweden (SE) from 1970 to 2020 with a particular attention to the possible contribution of mammography in survival improvements. These countries have organized health care largely in a similar way with an overriding principle that access to health services is offered with minimal direct costs to patients. Fairly similar mammographic screening programs were set up in each country but introduction and rolling out times differed to some extent, inviting for country-specific analyses. Even though we have no individual level data on a woman’s participation in the program, the attendance rates have generally been high, allowing a reasonable accuracy of designating population attendance. The Nordic cancer registries are the oldest national cancer registries in the world, and they cover practically all cancers without loss to follow-up [15]. The Nordic cancer registries have delivered aggregated data to the NORDCAN database which is now available at the International Agency for Cancer (IARC) web site (https://nordcan.iarc.fr/en/database#bloc2 (accessed on 15 August 2022)), enabling incidence, mortality, and survival studies through to 2020. We assessed changes in these parameters coincident with the implementation of the national mammographic services assuming that survival would increase several years past the introduction of mammography. Importantly, we assessed as a novel approach relative survival in breast cancer patients, irrespective of their cause of death, in addition to mortality which always measures cause-specific deaths [2,7,8,16].

## 2. Methods

The data used originate from the NORDCAN database which is a compilation of data from the Nordic cancer registries as described [15,17]. The database was accessed at the IARC website (https://nordcan.iarc.fr/en/database#bloc2 (accessed on 15 August 2022)). Relative 5- and 10-year survival data were available from 1970/1971 through 2019/2020 and the analysis was based on the cohort survival method for all but the last five-year period. For this, the hybrid analysis, combining period and cohort survival was used, as detailed [17,18]. Age-standardized relative survival was estimated using the Pohar Perme estimator [19]. Age-standardization was performed by weighting individual observations using external weights as defined at the IARC web site [20]. National general population life-tables stratified by sex, year, and age were used in the calculation of expected survival. Death certificate only cases were not included. Patients aged 90 years or older were excluded. Groups were analyzed if a minimum 30 patients were alive at the start and with a minimum 3 patients in any one of the age groups used for weights. Incidence and mortality analyses were also carried out on the NORDCAN database.

DK, FI, and NO defined the screened target population at 50–69 years, and with a 2-year screening interval [1,21]. The SE system is based on county definitions with many modest variations, including target age groups, starting at age 40 in 11 out of 21 counties and ending at age 74 in 10 in 21 counties. In some smaller counties, screening was started already in 1974 [1,5,22]. Participation rates have ranged from 60% to 90%, but these change with time [1,5,21]. In DK, mammography was introduced regionally starting between 1991 and 2008, and in each region the population was stepwise invited to attend [7]. In FI, the program was completed in a short time between 1986 and 1989 [1]. In NO, a staggered rollout was completed between 1996 and 2005 [8]. In SE, the completion was achieved between 1986 and 1997 [1]. To what extent opportunistic screening took place before the national rollouts is not known.

## 3. Results

The number of female breast cancers in year 1980 (before mammography) was 2257 in DK, 1649 in FI, 1605 in NO, and 4024 in SE. The cases diagnosed in age groups 50–69 years, targeted in mammography, accounted respectively for 45.3%, 45.0%, 44.2%, and 43.4% of all breast cancers in these countries. However, in many SE counties, a wider target age was applied.

**Figure 1:**
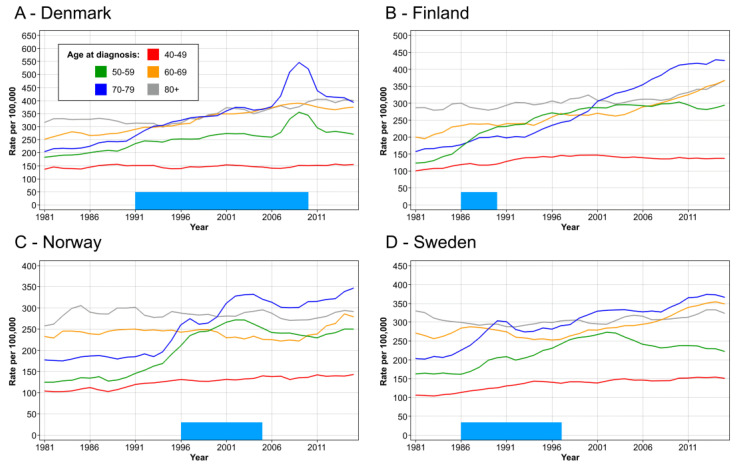
Age specific incidence for female breast cancer in DK (**A**), FI (**B**), NO (**C**) and SE (**D**). The bar in the bottom shows the approximate time of rolling out the mammographic screening to the national level.

Age specific incidence for female breast cancer is shown in Figure 1 for the Nordic countries. In DK, the incidence in age groups 50–59 and 60–69 increased modestly, but after 2006, in both age groups, there was a transient peak which disappeared at around 2011. The peak coincided with the implementation of the screening program in most of the country. In FI, incidence in the same age groups started to climb in 1986. For age group 60–69, it increased towards the end of the follow-up. For NO, the incidence in these two age groups showed a minor peak in 1997 and a prominent peak after year 2001. These coincided with the initial start of mammography in four counties in 1996 and the period when the rest of the country was covered by year 2005. In SE, there was a minor peak in these age groups at around 1990, and a more prominent peak at around 2001. 

**Figure 2:**
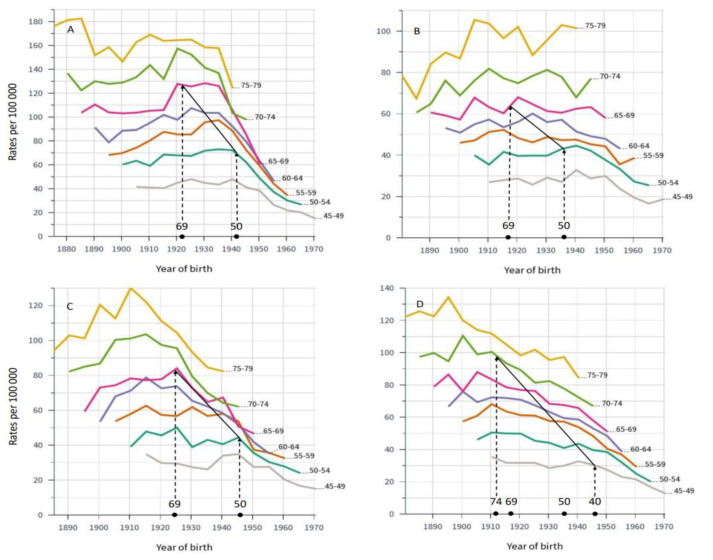
Age-specific mortality in breast cancer by births cohorts in DK (**A**), FI (**B**), NO (**C**) and SE (**D**). On top of *x*-axis birth time is marked for women eligible for screening in the particular countries according to the applied national screening ages. The solid diagonal line demarks the times when breast cancer mortality in the birth cohorts could first be affected by breast cancer screening, implemented between years 1991 and 2008 in DK, 1986–1989 in FI, 1996–2005 in NO and 1974–1986/1997 in SE. Mortality rates to the right of the solid line were generated when screening was available.

Mortality in breast cancer was analyzed by birth cohorts (Figure 2). On top of the *x*-axis, we marked the birth years of women when they were first eligible for screening, having reached age 50 or 69 years (wider county-dependent age range in SE). In DK, screening was started in 1991 among 50–69-year old women covering birth cohorts from 1922 to 1941. Morality started to decline in the screening-age birth cohorts, particularly in diagnostic group 65–70 years, but also in the older diagnostic groups. Morality declined least in diagnostic age group 45 to 49 years, not eligible for screening. This was consistent in all countries, even in SE where some counties started screening at age 40 years. Notably, FI with much lower death rates than the other countries showed only a modest decrease in mortality in the screening-age groups and no improvement in the two oldest age groups. In SE, mortality declined in the oldest age groups well before screening was started. 

**Figure 3:**
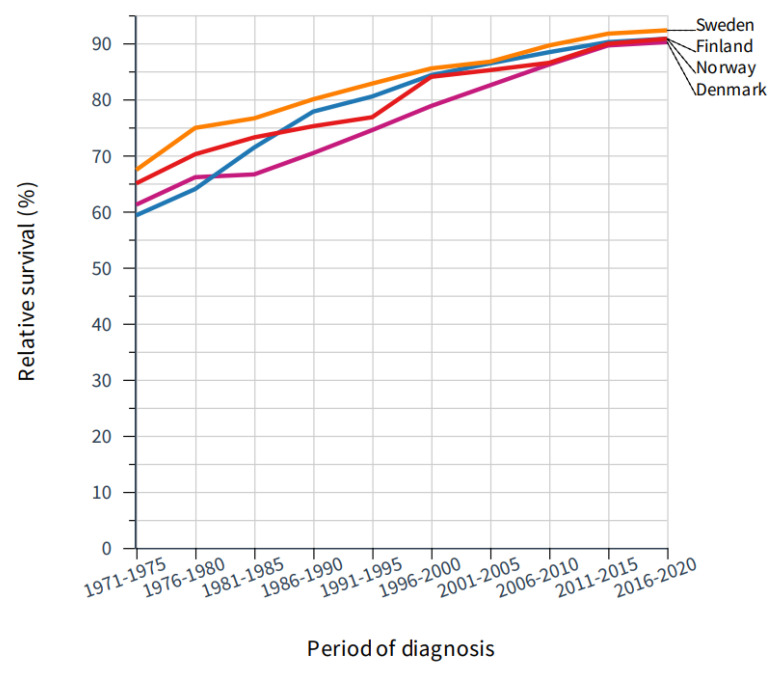
Improvement in 5-year relative survival in female breast cancer in the Nordic countries through 50 years.

Relative 5-year survival in the critical period for mammography rollout is shown in Figure 3 and the underlying data (including also 10-year survival) since 1971 are tabulated in Table 1. Survival was best for SE, and a significant increase in 5-year survival took place during the implementation of mammography (1986–1997, Table 1). However, a steep increase in survival took place even before this period. In FI, survival improved from 71.4% in the pre-mammography period to 77.8% and 80.5% in the start of the mammography period, but improvement was marked even before that period. In DK, large improvements in survival coincided with the rollout of mammography, while in NO a significant increase in 5-year survival was noted only for years 1996–2000 and for 2011–2015.

**Figure 4:**
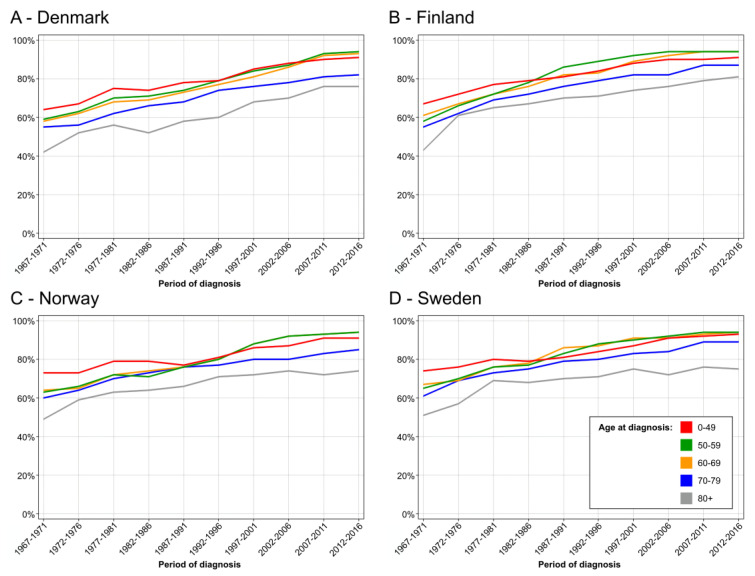
Age specific 5-year relative survival in female breast from 1967–71 to 2012–16, DK (**A**), FI (**B**), NO (**C**) and SE (**D**).

Age-specific five-year relative survival data on breast cancer showed an increase in survival in all age groups but we wanted to compare age groups 50–69 against others after the introduced mammography (Figure 4). In DK, survival in these age groups improved marginally better than in other age groups from 1992–1996 onwards, and in the last 10-year periods they showed the best survival (Figure 4A). The differences were clear in 10-year survival (Figure S1). In FI, survival in age group 50–59 developed very well and from 1987–1991 onwards it showed the best survival. Yet, age-group 60–69 caught up in the last 10 years (Figure 4B). The superior survival in FI age groups 50–69 years compared to other age groups is very clear in 10-year survival in Figure S2. NO data are essentially similar to FI, and survival in age groups 50–59 and 60–69 is superimposable in 1992–1996 (Figure 4C), and supported by 10-year survival in Figure S3. The SE data show a relatively good survival in the youngest age group which almost matched survival in age groups 50–69 (Figure 4D and Figure S4).

## 4. Discussion

We assessed here survival trends in breast cancer around the time of introduction of national mammographic screening in the Nordic countries. To our knowledge, survival studies have not been used to evaluate the impact of mammography, and no such studies are cited in the IARC handbook on breast cancer screening [1]. The analysis is ecological as we had no individual data on women’s screening attendance, and thus the findings are at most suggestive of trend changes due to mammography. The previous studies from the Nordic countries and elsewhere assessing the possible reduction of breast cancer mortality due to screening have remained controversial and we have no opportunity or scientific justification to enter this vast discussion here [4,5,6,7,8,9,23]. Screening would be expected to preferentially detect early tumors (stage migration), which as such would lead to increased survival (lead-time bias), which we could not correct [1]. Although a decrease in mortality would be expected to increase survival, the present metric of relative survival does not measure breast cancer specific deaths but any deaths in breast cancer patients [24]. Causes of death in breast cancer patients are diverse and may be ambiguously related to this cancer after many years of survival [16,25]. Thus, relative survival may be less sensitive to bias compared to cause-specific mortality. However, both measures would be sensitive to overdiagnosis [23]. In the context of screening the agnostic metric, relative survival has the advantage of not being sensitive to factors that relate to women’s attendance to breast cancer screening; such factors may bias comparisons between outcomes in screened and unscreened women. A further weakness in the present analysis is that we could not statistically assess age group specific differences in survival trends because these metrics were not available in NORDCAN.

Our analysis of incidence trends revealed a relative increase in incidence in the screened age groups compared those not screened as has been reported elsewhere for SE [5]. The relationship to screening was most pervasive in DK and NO (Figure 1) as the transient incidence peaks coincided with rolling out of the main national screening activities [7,8]. The analysis of mortality in breast cancer showed a decrease in mortality in birth cohorts eligible for screening but even in older birth cohorts (Figure 2). Perhaps the most consistent finding possible related to screening was that the mortality decline was smallest in age group 40–45 years, not eligible for screening, except in some SE counties. We showed that survival increased in all countries after introduction of mammographic services, but survival was developing well also in pre-mammography era for other reasons perhaps related to improving treatment. Survival improvements in breast cancer have often been assigned to treatment for which surgery has been the main therapeutic modality, supported by radiotherapy [26]. Adjuvant therapies have had a major impact on survival, and their indications have been extended and novel agents have been taken to use [26]. Chemotherapy is increasingly used in metastatic and recurrent breast cancer [27]. Additionally, greater awareness of breast cancer which, together with novel imaging techniques, would favor early detection (Figure 3 and Table 1) [14]. However, when 5-year survival analysis was carried out by age groups, the superior survival in the screened age group vs. unscreened women was observed several years after rolling out of mammographic activities in all countries (Figure 4). The differences between screened and unscreened women were even clearer when 10-year survival was assessed (Figures S1–S4). As indicated above, we had no means of assessing the differences statistically, but consistency between the four countries adds weight to the observation.

## 5. Conclusions

The novel results on relative survival of breast cancer patients are not inconsistent with the benefits of mammography. With the caveat of an ecological study, i.e., screening-related lead-time bias and limited statistical confirmation, the descriptive data suggest that survival in breast cancer was improved in the screened age groups of the Nordic women compared to their unscreened mates. In spite of the ecological weakness of the study, it has strengths, including the validity of cancer data, well documented target groups for mammographic screening, and the high participation rates. It is difficult, or even impossible, to quantify the contribution of various therapeutic modalities to survival in breast cancer at the population level, but the present results suggest that mammography needs to be considered as a likely contributor.

## Figures and Tables

**Table 1:** Breast cancer relative survival (%, [95% confidence interval]) in the Nordic countries.

Period	5-Year Survival				10-Year Survival			
	Denmark	Finland	Norway	Sweden	Denmark	Finland	Norway	Sweden
1971–1975	61.2 [59.8–62.7]	59.3 [57.3–61.3]	65.0 [63.3–66.8]	67.4 [66.4–68.4]	45.8 [43.8–47.9]	41.6 [39.3–44.0]	51.1 [48.6–53.8]	52.6 [51.1–54.2]
1976–1980	66.1 [64.8–67.3] *	64.0 [62.5–65.5] *	70.2 [68.6–71.7] *	74.9 [74.0–75.8] *	50.5 [48.7–52.4] *	46.8 [44.8–48.9] *	55.4 [53.0–57.8]	61.6 [60.1–63.1] *
1981–1985	66.6 [65.5–67.8]	71.4 [70.0–72.8] *	73.2 [71.8–74.6] *	76.6 [75.7–77.5]	52.0 [50.3–53.8]	58.7 [56.5–60.8] *	59.2 [57.1–61.4]	63.5 [62.1–64.9]
1986–1990	70.4 [69.3–71.5] *	77.8 [76.5–79.1] *	75.2 [73.9–76.5]	80.0 [79.2–80.7] *	57.5 [55.8–59.3] *	67.6 [65.5–69.7] *	62.2 [60.2–64.3]	69.5 [68.3–70.8] *
1991–1995	74.5 [73.4–75.5] *	80.5 [79.3–81.7] *	76.8 [75.6–78.1]	82.8 [82.1–83.6] *	63.8 [62.1–65.6] *	70.2 [68.2–72.2]	65.7 [63.8–67.7]	74.4 [73.2–75.7] *
1996–2000	78.8 [77.8–79.8] *	84.3 [83.3–85.3] *	84.0 [82.9–85.1] *	85.5 [84.8–86.2] *	71.1 [69.4–72.8] *	75.0 [73.1–76.9] *	75.2 [73.4–77.1] *	77.6 [76.4–78.7] *
2001–2005	82.5 [81.6–83.4] *	86.4 [85.5–87.4] *	85.2 [84.2–86.3]	86.7 [86.1–87.4]	75.7 [74.1–77.3] *	79.8 [78.2–81.4] *	76.2 [74.5–78.0]	80.6 [79.5–81.7] *
2006–2010	86.2 [85.4–87.0] *	88.4 [87.6–89.2] *	86.5 [85.5–87.6]	89.6 [89.0–90.2] *	81.5 [80.0–83.0] *	83.8 [82.4–85.3] *	79.6 [77.9–81.3]	83.9 [82.9–85.0] *
2011–2015	89.6 [88.9–90.4] *	90.2 [89.5–90.9] *	89.9 [89.0–90.8] *	91.7 [91.1–92.2] *	86.3 [85.0–87.7] *	86.1 [84.8–87.4]	84.0 [82.3–85.8] *	87.2 [86.1–88.2] *
2016–2020	90.2 [89.5–90.9]	90.8 [90.2–91.5]	90.8 [90.0–91.7]	92.3 [91.7–92.8]	86.9 [85.7–88.2]	86.6 [85.5–87.8]	84.9 [83.1–86.7]	87.8 [86.8–88.8]

* Non-overlapping 95%CIs between this and the previous period.

## Data Availability

Publicly available NORDCAN data can be accessed at (https://NORDCAN.iarc.fr/en/database#bloc2 (accessed on 15 August 2022)).

## Supplementary Materials

The following supporting information can be downloaded at: https://www.mdpi.com/article/10.3390/cancers14235907/s1, Figure S1: Age specific 10-year relative survival in female breast in Denmark from 1967–1971 to 2012–2016; Figure S2: Age specific 10-year relative survival in female breast in Finland from 1967–1971 to 2012–2016: Figure S3: Age specific 10-year relative survival in female breast in Norway from 1967–1971 to 2012–2016; Figure S4: Age specific 10-year relative survival in female breast in Sweden from 1967–1971 to 2012–2016.
