# Supplementary material for: Incidence, Mortality and Survival Trends in Breast Cancers Coincident with Introduction of Mammography in the Nordic Countries
Source: Cancers (Basel). 2022 Nov 29;14(23):5907. doi: 10.3390/cancers14235907 (PMC9736677; doi:10.3390/cancers14235907)

Figure S1. Age specific 10-year relative survival in female breast in DK from 1967-71 to 2012-16.

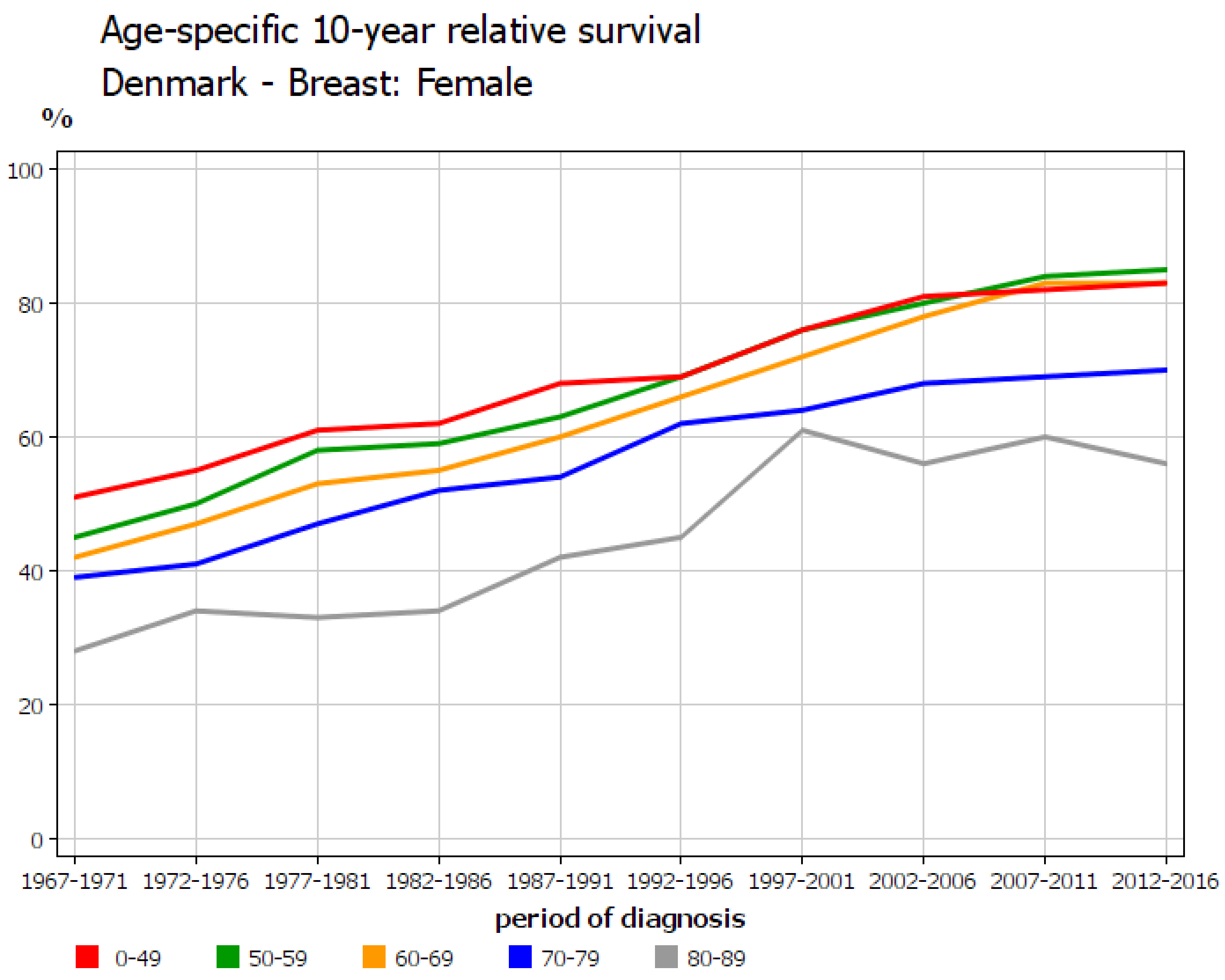

Figure S2. Age specific 10-year relative survival in female breast in FI from 1967-71 to 2012-16.

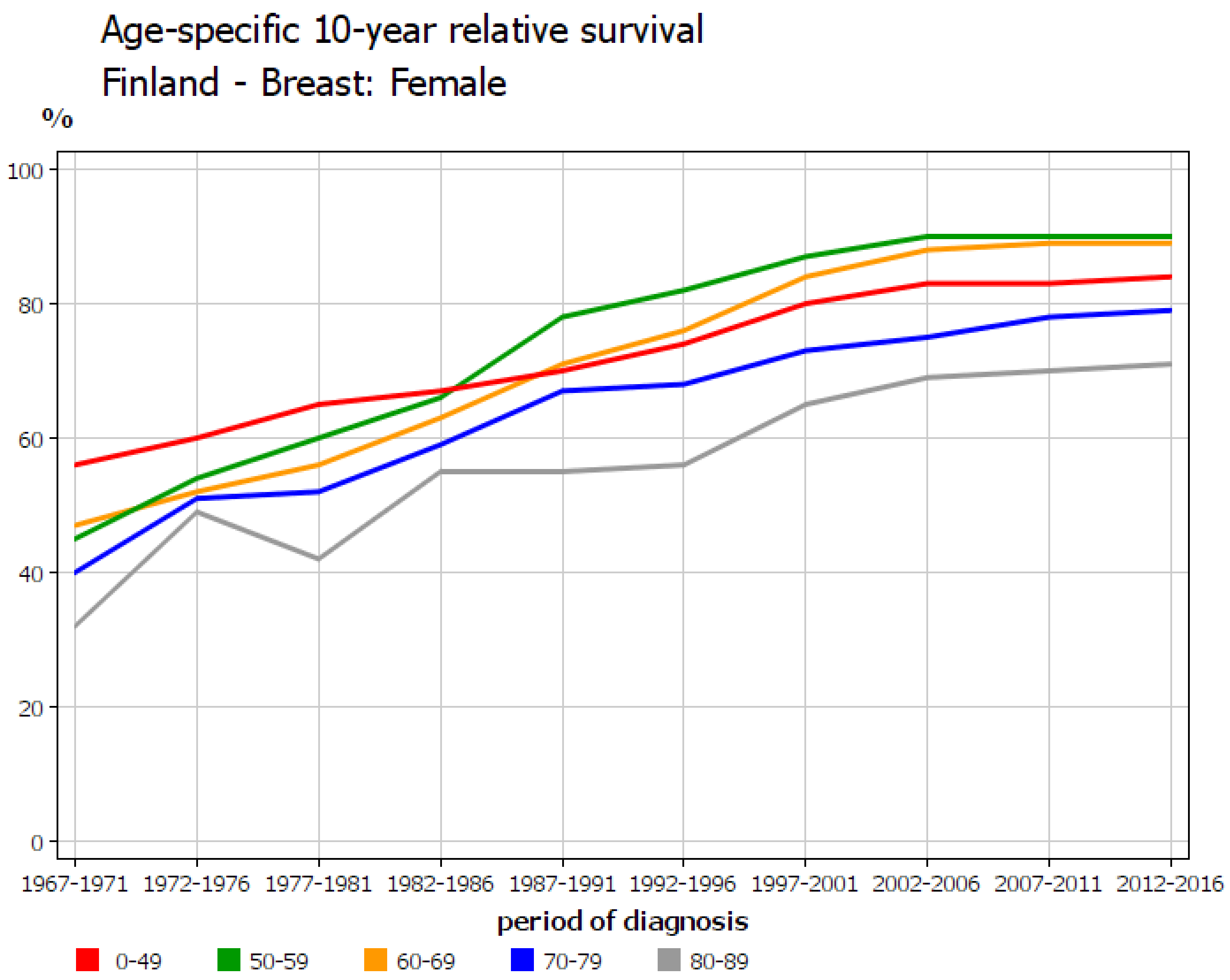

Figure S3. Age specific 10-year relative survival in female breast in NO from 1967-71 to 2012-16.

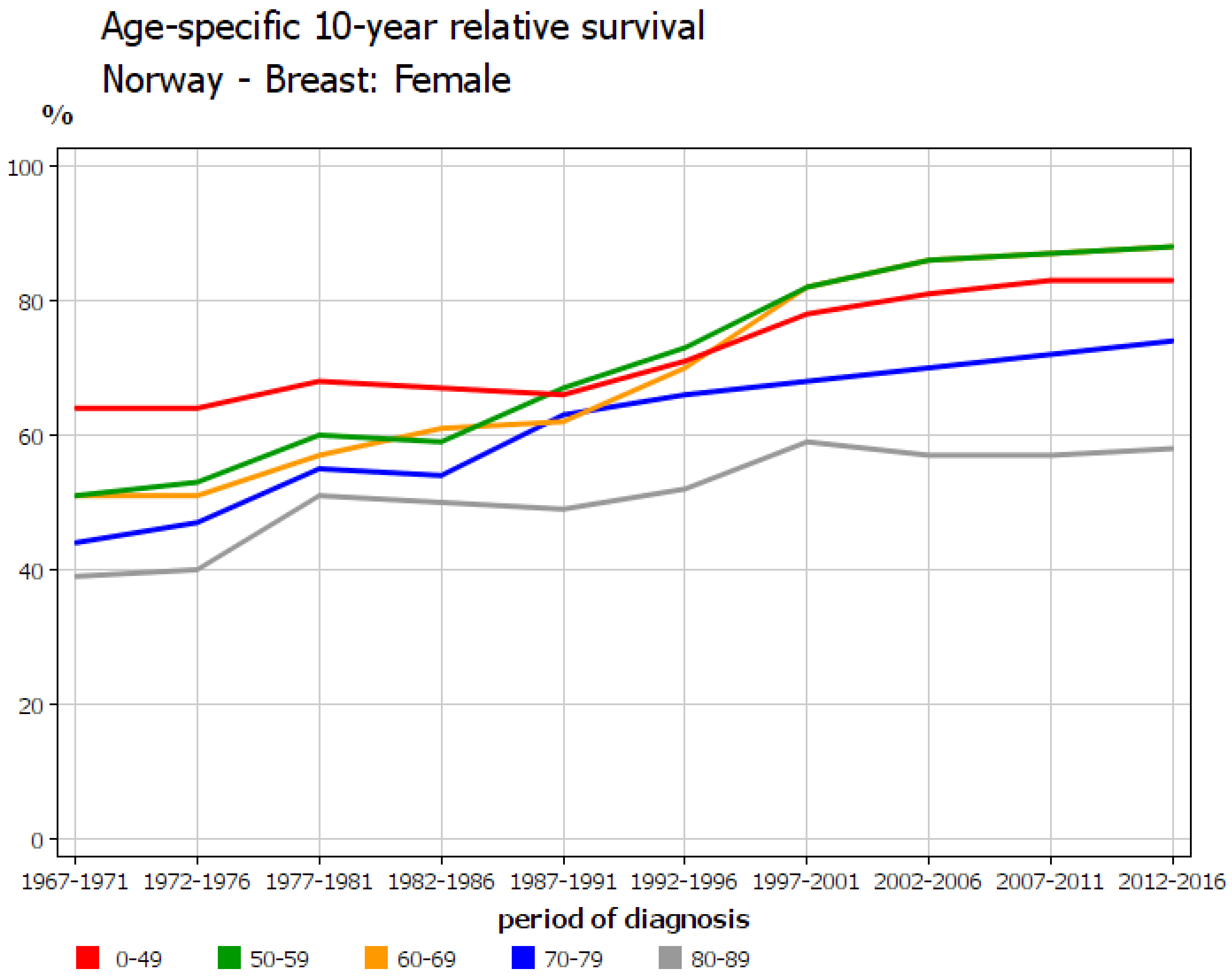

Figure S4. Age specific 10-year relative survival in female breast in SE from 1967-71 to 2012-16.

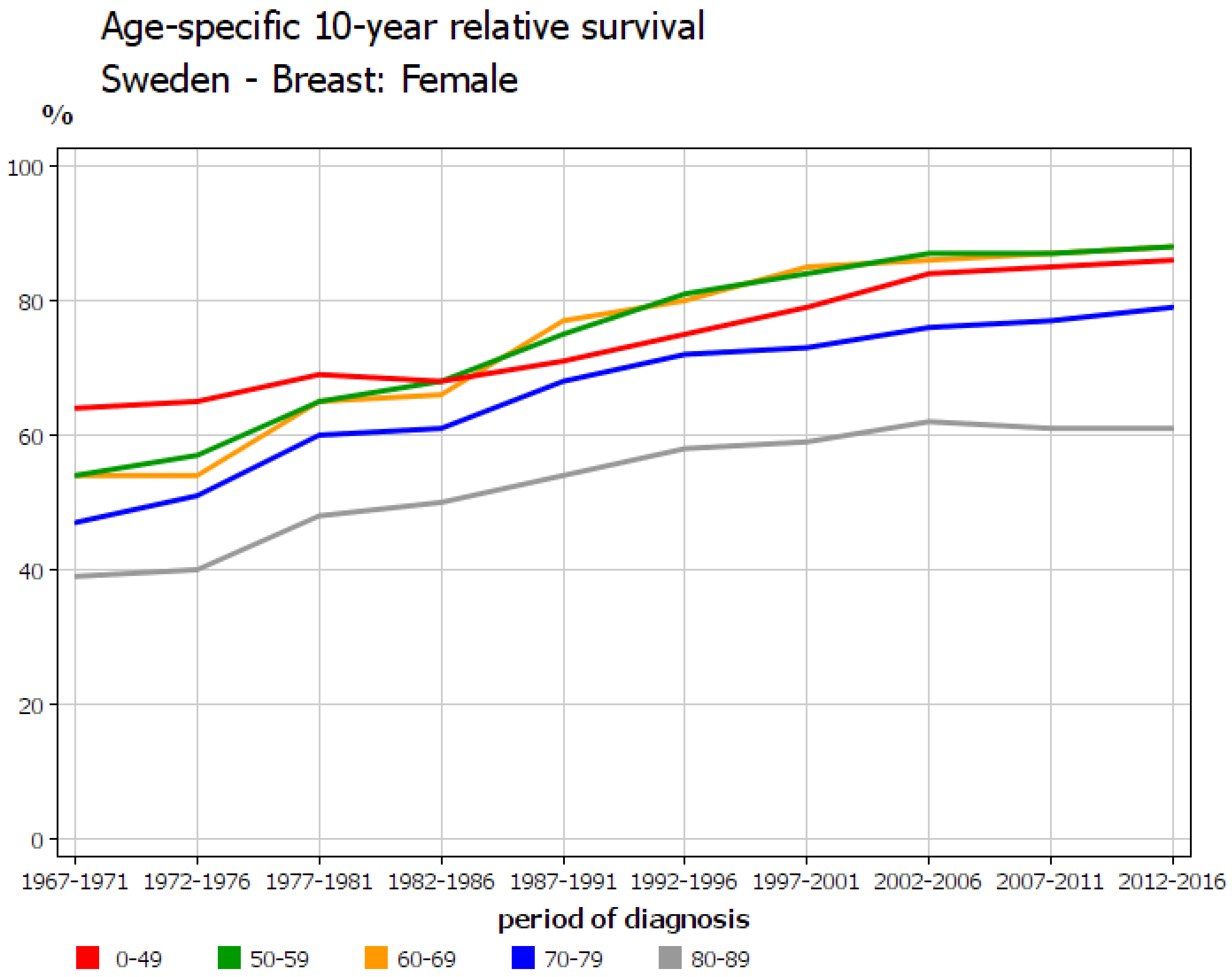

Supplement: Supplementary file 1 [file cancers-14-05907-s001.zip › cancers-2042816-supplementary.pdf]
